# Finding microbial composition and biological processes as predictive signature to access the ongoing status of mangrove preservation

**DOI:** 10.1007/s10123-024-00492-z

**Published:** 2024-02-22

**Authors:** Fabíola Marques de Carvalho, Marcele Laux, Luciane Prioli Ciapina, Alexandra Lehmkuhl Gerber, Ana Paula C. Guimarães, Vinícius Prata Kloh, Moacir Apolinário, Jorge Eduardo Santos Paes, Célio Roberto Jonck, Ana Tereza R. de Vasconcelos

**Affiliations:** 1 Laboratório de Bioinformática, Laboratório Nacional de Computação Científica, Avenida Getúlio Vargas 333, Quitandinha Petrópolis, Rio de Janeiro, 25651-075 Brazil; 2 Petróleo Brasileiro S. A., Centro de Pesquisa Leopoldo Américo Miguez de Mello, Rio de Janeiro, RJ Brazil

**Keywords:** Metagenome, Microbiome, Differential abundance, Metabolism, Mangrove preservation

## Abstract

Mangroves are complex land-sea transition ecosystems whose microbiota are essential for their nutrient recycling and conservation. Brazil is the third-largest estuarine area in the world and “Baía de Todos os Santos” (BTS) is one of the largest bays of the country, with wide anthropogenic exploration. Using a metagenomic approach, we investigated composition and functional adaptability as signatures of the microbiome of pristine and anthropized areas of BTS, including those under petroleum refinery influence. The taxonomic analysis showed dominance of sulfate-reducing Desulfobacteraceae, Rhodobacteraceae, and Flavobacteriaceae. Taxa were significantly diverse between pristine and disturbed areas. Disturbed mangroves showed a notary increase in abundance of halophilic, sulfur-related, and hydrocarbon-degrading genera and a decrease in diatoms compared to pristine area. The metabolic profile of BTS mangroves was correlated with the differentially abundant microbiota. Two ecological scenarios were observed: one marked by functions of central metabolism associated with biomass degradation and another by mechanisms of microbial adaptability to pollution conditions and environmental degradation. Part of the microbiome was distinct and not abundant in Brazilian estuarine soils. The microbiome signature observed in each BTS mangrove reflects how human actions impact the diversity of these ecosystems and also emphasize their role in attempting to restore disturbed mangroves. The microbiome may act as a potential biological indicator of the preservation status of these soils, despite the limitation of soil property conditions. Additionally, our data pointed to metagenomics as an additional tool for environmental assessment and reinforced the need for protective measures for the mangroves under study.

**Supplementary Information:**

The online version contains supplementary material available at 10.1007/s10123-024-00492-z.

## Introduction

Mangroves are intertidal ecosystems of worldwide distribution in tropical and subtropical coastlines (Giri et al. 2011). These ecosystems have high primary productivity and are important for climate change mitigation due to their large capacity for biogeochemical nutrient cycling and increased carbon storage capacity (Alongi 2002, 2014; Bouillon et al. 2008), besides providing environmental conditions suitable to the development of marine species and contributing to area preservation against erosion and natural disasters (Palit et al. 2022). Also, they play a major role in energy and mineral cycles along tropical coasts, acting as a biogeochemical barrier to trace metal transport and a nutrient source for microorganisms, plants, and animals (Freitas et al. 2002). Regardless of their ecological importance, 35% of the global mangrove areas have been lost over the last 20 years (Valiela et al. 2001) as a consequence of strong anthropogenic pressures (Duke et al. 2007; Spalding et al. 2010; Hamilton and Casey 2016), impacting the benthic biodiversity and leading to a drastic decline of microbially mediated decomposition rates (Carugati et al. 2018).

Mangroves cover 9900 km^2^ of Brazilian territory, the country with the third-largest estuarine area (Giri et al. 2011), comprising 8.5% of the global mangrove area (Diniz et al. 2019). The “Baía de Todos os Santos” (BTS) is the largest bay in Brazil and the second largest in the world (IBGE 2019). Located in Northeastern Brazil, around the 8th major metropolitan area of the country (Salvador city, Bahia State), BTS has a maximum size of 1223 km^2^ and an average depth of 9.8 m. The intertidal area corresponds to 27% of the maximum area of the BTS (327 km^2^), most of which is occupied by mangroves and non-vegetable regions of varied sediment textures (Cirano and Lessa 2007). BTS holds the largest petrochemical settlement in the southern hemisphere and its maritime activity represents almost 5% of the annual flow throughout Brazil. The offshore oil and gas fields located 100 km from the bay (ANP 2017) impact the continental shelf, located northeast of the bay, through the receipt of biochemical drainage and sewage (Cirano and Lessa 2007). In addition to oil exploration, areas of the Baía de Todos os Santos have been used for the extraction of calcareous sands, fishing activity, and intensive tourism (Leao and Dominguez 2000). Moreover, a study of chemical monitoring of saline water and sediments at Aratu harbor over 2 years (2010–2012) indicated the bioaccumulation of environmental contaminants copper and lead metals in sediments of BTS (Rocha et al. 2016a, b).

Studies of microbial communities of mangroves have been highlighted due to their close nutrient-plant relationship, which functions as a mechanism to recycle and conserve nutrients essential for the productivity, conservation, and rehabilitation of these ecosystems (Holguin et al. 2001; Jansson and Hofmockel 2018). In this context, metagenomics and 16S rRNA high-throughput sequencing methodologies have been employed to evaluate the taxonomic and functional diversity of mangroves (Allard et al. 2020; Nóbrega et al. 2022), as well as community changes resulting from environmental, anthropogenic, and industry actions around the world (Andreote et al. 2012; Cabral et al. 2016; Huergo et al. 2018; Li et al. 2019; Liao et al. 2020; Mai et al. 2021).

The microbiome and their potential metabolic properties in Brazilian mangroves have been poorly characterized (40 public references on The National Center for Biotechnology Information, January 2024) and widely focused on North, Southeast, and Northeast regions, mainly in Amazonas, São Paulo, and Ceará States, respectively (Andreote et al. 2012; Nogueira et al. 2015; Mendes and Tsai 2018; Cotta et al. 2019; Nóbrega et al. 2022; Costa et al. 2023). In Amazonian mangroves, microbial groups driving carbon, nitrogen, methane, and mainly sulfur cycles and that are consistently found across pristine areas (Nóbrega et al. 2022) suffer a considerable reduction of diversity in areas under human impact (Costa et al. 2023). In Southeastern Brazil, the microbial communities of degraded mangroves are structured orderly, adapting to new conditions and establishing a functional balance without loss or impairment of the ecological system (Andreote et al. 2012; Cotta et al. 2019). In addition, these soils have an elevated concentration of heavy metals, originating from pollution by petroleum, sludge, and other urban waste, with members of Gammaproteobacteria and Deltaproteobacteria classes contributing to the detoxification processes (Cabral et al. 2016). Sanders et al. (2014) observed a rapid accumulation of organic carbon and total nitrogen in these ecosystems due to increased organic matter originating from phytoplankton, benthic algae, or other autochthonous and allochthonous sources, which can lead to coastal eutrophication. Similarly to Southeastern, in Paranaguá Bay (Southern region, Paraná State, Brazil), metabolic functions are more conserved than microbial structure, clustered according to biome type over the salinity gradient (Ceccon et al. 2019).

In the Brazilian Northeastern, the microbiome of anthropized mangroves from Ceará State varied in response to a semiarid climate with altered Proteobacterial class abundance, possibly related to eutrophication. However, they did not metabolically differ from the pristine ecosystem (Nogueira et al. 2015). Minimum temperature, precipitation, organic carbon, and evapotranspiration were the main variations in the diversity of the northeastern semiarid microbiomes. Furthermore, the resilience of these communities to cope with adverse conditions may be the factor triggering functional mangrove adaptability (Tavares et al. 2021).

In the Bahia State Brazil, the bioavailability of organic and toxic compounds affecting the geochemical cycles in mangroves has been explored (Paixão et al. 2011; Silva et al. 2014; Bomfim et al. 2015; Paes et al. 2022), but very little attention has been directed to the microbial communities inhabiting the BTS.

This metagenomic study aims to identify microbial and metabolic signatures (i.e., singular microbial community structure and functional diversity) of conserved, anthropized, and oil refinery estuaries along the BTS under influence of human and industrial activities as a possible assessment measure tool of the preservation degree from these areas.

## Material and methods

### Study sites and sample collection

**Fig. 1:**
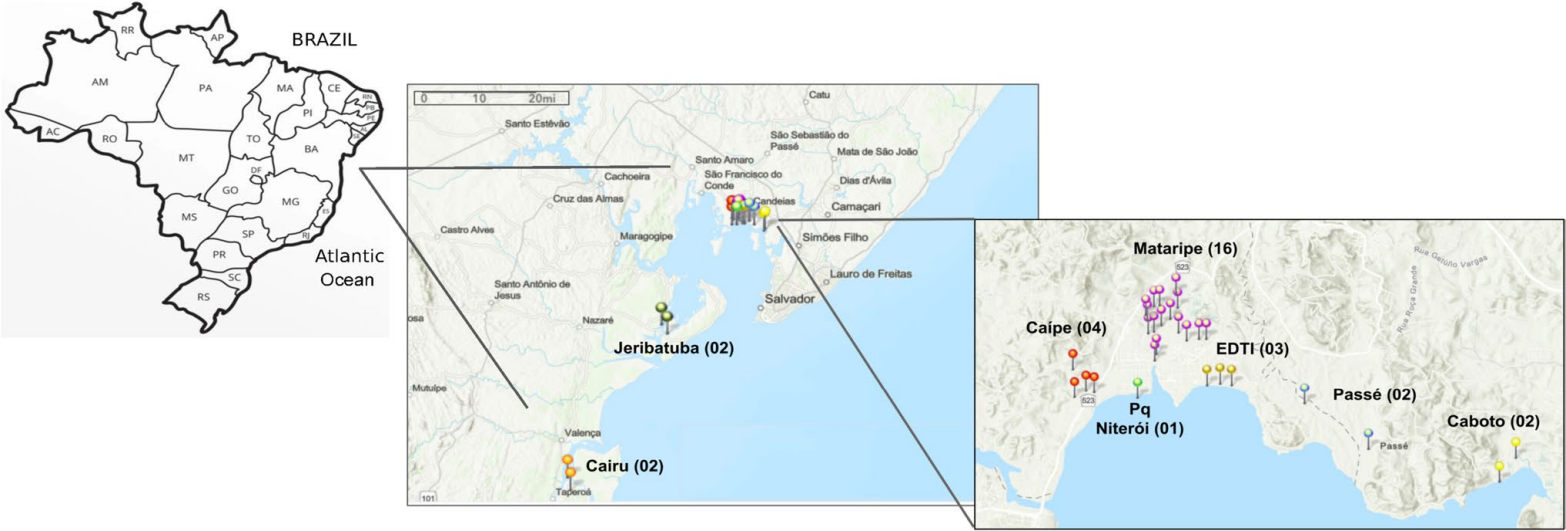
Geographic location of Baía de Todos os Santos (BTS) mangroves sampled in this study, located around Salvador City, Bahia State, Brazil. Eight mangrove regions were selected, and the number of points analyzed in each region is shown in parentheses. Points of Cairu were named as CAM 01 up to 02; Jeribatuba as JER 01 up to 02; Parque Niterói as NIT 01; Caípe as CAI 01 up to 04; Mataripe as MAT 01 up to 16; ETDI as ETD 01 up to 03; Passé as PAS 01 up to 02; and Caboto as CBT 01 up to 02. For each point, three samples were collected

The study covered eight distinct mangrove regions of the Baía de Todos os Santos (BTS), an inlet of the Brazilian coast in Bahia (Fig. 1 and Supplementary Table 1). A total of 96 mangrove sediment samples were collected from regions inside and outside the BTS, including the surroundings and inside the refinery. The samples were obtained in triplicate for each one of the 32 points, being Caípe (04), Mataripe (16), ETDI (03), Parque Niterói (01), Caboto (02), Passé (02), Jeribatuba (02), and Cairu (02) (Supplementary Table 1). The Caípe (CAI) and Mataripe (MAT) regions correspond to the rivers Caípe and Mataripe, respectively, which flow into the BTS and cross an oil refinery. Caípe River borders the refinery, while the Mataripe River runs through the middle of the refinery, which justifies the largest number of sampling points in this region. The ETDI (ETDI) and Parque Niterói (Pq Niterói—NIT) regions correspond to two areas inside the refinery. ETDI is an effluent treatment station where the current mangrove has been restored. However, it still has very different characteristics from a typical mangrove. In this area, the sediment was considerably finer and more profound than other BTS sampling sites, making access impossible without the aid of supports, which was not necessary at any other sampling points. Organoleptic characteristics were also markedly different from those found in other mangroves, even those under the direct influence of the refinery. Parque Niterói (NIT) is an area where the mangrove restoration has not been successful. Caboto (CBT) and Passé (PAS) areas are near the refinery and although not under its direct influence are impacted by other types of anthropogenic pressures such as other industries and human occupation. Jeribatuba (JER) and Cairu (CAM) are located in preserved areas away from great anthropic pressures and are considered reference regions within and outside the BTS, respectively.

Sediments were collected on the first 10 days of October 2019 at low tide at 0 to 2 cm depth. Soil samples were collected with a sterilized stainless steel spatula, with the collector wearing a face mask and nitrile gloves. Soils were placed in RNase-free Falcon tubes and kept on ice (4 °C) until arrival at the field base. There, they were frozen at − 20 °C and later transported on dry ice to the laboratory, where they were stored in an ultrafreezer (− 80 °C) until processing.

### DNA extraction and whole genome shotgun sequencing

Total DNA extraction was performed using the PowerSoil® DNA Isolation Kit (Mobio Labs, Inc., Solana Beach, CA, USA) according to the manufacturer’s guideline at SENAI Institute of Innovation in Biosynthetic and Fibers (SENAI CETIQT, Rio de Janeiro, RJ, Brazil). The quantitative and qualitative DNA analysis and metagenomic library preparation and sequencing were performed at Computational Genomics Unity Darcy Fontoura de Almeida (UGCDFA) of the National Laboratory of Scientific Computation (LNCC) (Petrópolis, RJ, Brazil). The DNA concentration and integrity were determined using Agilent TapeStation 4200 System (Agilent Technologies, USA) with Genomic DNA ScreenTape according to the manufacturer’s instructions. Libraries were constructed using the Nextera DNA Flex library preparation kit (Illumina, USA) according to the manufacturer’s recommendations. Sequencing was conducted on an Illumina NextSeq 500 platform (Illumina, San Diego, CA, USA) using the NextSeq 500/550 high-output kit v2.5 (Illumina, USA), with the system set to produce 2 × 150-bp reads.

### Bioinformatic processing and statistical analyses

Raw reads were submitted to BBduk (BBMap software v.38.81 [https://github.com/BioInfoTools/BBMap]) for quality control (i.e., the identification and filtering of low-quality reads and sequencing artifacts). Reads with a quality threshold lower than a Phred score of 20 (with a sliding window of 10 bases) and a length smaller than 50 bp, Illumina adapters, and phiX174 were removed using the following parameters: minlength = 50, mink = 8, qout = auto, hdist = 1, *k* = 31, trimq = 10, qtrim = rl, ktrim = l, minavgquality = 20, and statscolumns = 5.

The high-quality reads were then submitted to taxonomic classification using the Kaiju 1.7.3 software (Menzel et al. 2016) and the NR_EUK database (February 2021 version). It is known that Kaiju specificity and accuracy are dependent on the coverage and taxa abundance, with limitations for more precise species-level inferences (Ye et al. 2019). Nevertheless, in simulations where Critical Assessment of Metagenome Interpretation (CAMI) datasets (i.e., short DNA sequencing reads, comprised of different taxon profiles, with only 30–40% of reads are simulated from known taxa—Sczyrba et al. 2017) were used at the genus level, Kaiju had an improvement on precision-recall curve (AUPR) scores similarly to those observed to the other classifiers (Ye et al. 2019). Thus, Kaiju was used to capture partial genomes, in a broader overview of the environmental diversity of sampling mangroves.

The Chao1 estimator investigated microbial richness, and Shannon and Simpson indexes were applied to compute the diversity under the absolute abundance of bacterial species. Richness and diversity indices were obtained using the skbio.diversity.alpha_diversity function of a Python script written in the skbio package (scikit-bio Development Team. 2020. scikit-bio: a bioinformatics library for data scientists, students, and developers, version 0.5.5). Alpha diversity indices were sampled graphically using the vegan package (version 2.6–4, R Core Team 2021). The diversity of each point was compared pairwise using ANOVA, with Tukey’s post hoc test (ggpplot2 package, version 3.4.1, R Core Team 2021), considering a value of *p* < 0.05 for statistical significance. Principal coordinate analysis (PcoA), generated to determine the distances or dissimilarities between the structures of the microbial communities, was obtained using the NMDS method and Bray–Curtis metric available in the Phyloseq package (McMurdie and Holmes 2013)(version 1.40.0, R Core Team 2021).

Nonrandom library size normalization corrected sequencing depth variations among samples to make the samples comparable. For this, a factor reflecting each sample-specific library size was applied to the respective read counts [calculated as factor = (n trim reads ss/n trim reads sls) × OTU reads ss, where ss is the specific sample, sls is the smallest library sample size across all samples, and OTU is the operational taxonomic units]. The microbial composition was investigated by first analyzing representative taxa (for phylum, family, genus, and species levels) and, in more detail, those that were differentially abundant (for family and genus levels). The first one was restricted to the 15 taxa with the highest abundance for each sampling point. The entire set of taxa identified for the taxonomic level was evaluated to analyze differential abundance. Sequences of prokaryotes, eukaryotes, archaea, and viruses were maintained in the analyses to broadly characterize the diversity found in the sampled mangroves, as well as identify taxa other than bacterial that could contribute as members differentially present in a specific area. The difference in microbial composition abundance among the sampling points was obtained using the ANCOM-BC package (Lin and Peddada 2020) (version 1.6.4, R Core Team 2021). Taxa with qualitative differential abundance were selected using FDR for *p*-value false-positive adjustment and significance of *p* < 0.05. The Jvenn tool (Bardou et al. 2014) was used to identify a region’s common and/or unique taxa.

For the metabolic inference analyses, the reads were assembled into contigs using Metaspades software (version 3.14) (Nurk et al. 2017), and parameter “-k 21,33,55,77.” Only contigs larger than 500 bp were included in the subsequent analyses. The contigs were then submitted to Prodigal software (v. 2.6.3) (Hyatt et al. 2010) for prediction of gene coding sequences (or “Open reading frames,” ORFs), applying the -g 1 -p meta options. Annotation and alignments were conducted with ORFs longer than 50 amino acids against the EggNOG database (Huerta-Cepas et al. 2019) (EggNOG mapper version 2.0.8 Feb 2021), which also compiles additional information from Cluster of Orthologous Genes (COG) (Tatusov et al. 2000). The following parameters were applied for the alignment: e-value ≤ 1e-5, identity > 60%, and query/subject coverage > 60%. The functional analyses were performed to identify metabolic categories associated with the spatial distribution of sampling points and correlate them with their taxonomic composition. To investigate whether the variance of the functional matrices was correlated to the spatial distribution of the samples, a permutational multivariate analysis of variance was conducted using distance matrices and the “adonis2” function from the Vegan package (Oksanen et al. 2022)(version 2.6–4, R Core Team 2021). The dispersion among the triplicates of each sample point was verified by a multivariate analysis of homogeneity of group dispersion using the “betadisper” function from the Vegan package (version 2.6–4, R Core Team 2021). Since high heterogeneity was observed among the triplicates of each point, the matrices were clustered using the “*K*-means clustering” method to minimize the internal variation compared to the variability between clusters. The *K*-means clustering was based on EggNOG (clusterEgg) matrices for the region and sampled point. To identify functions that best distinguished sample clusters from the EggNOG matrices, supervised classification models generated by recursive partitioning and regression trees were used (Dinsdale et al. 2013). Initially, a series of test/validation partitions were created using the createDataPartition function from the Caret package (version 6.0–93, R Core Team 2021). Then, the train function was used to evaluate the parameters and performance of different classification and regression methods (lda—linear discriminant analysis; rpart—recursive partitioning and regression training; knn—K nearest neighbors, and rf—random forest). Metabolic functions of the highest importance for each sample cluster were identified using the random forest method (Breiman 2001), according to accuracy and Gini metrics (Menze et al. 2009). The specificity and predictive power metrics were evaluated using the predict and confusion matrix functions. Regression trees were generated using the “rpart” function (rpart package, version 4.1–19, R Core Team 2021), obtaining a final model with minimal redundancy risk (Breiman et al. 1984).

The variables highlighted by the classification methods were additionally analyzed in multivariate ordination models (Dinsdale et al. 2013). Redundancy analysis (RDA), by function “rda” (Vegan package, version 2.6–4, R Core Team 2021), was adopted to investigate which functional categories were correlated to the spatial distribution of samples and taxonomic composition. The “ordistep” function (Blanchet et al. 2008) (Vegan package, version 2.6–4, R Core Team 2021) was adopted to determine the metabolic functions and taxa significantly related to the matrices distribution using permutational tests. All data were scaled to a single variance for the ordination analyses, thus defining a correlation analysis between the variables. The functional and taxonomic data matrices used a relative abundance of hits. The overall significance of the model was checked using ANOVA (*p* < 0.05) and the significance of the canonical axes was checked with the “permutest” function (Vegan package, version 2.6–4, R Core Team 2021).

The metabolic functions identified by the classification models, together with the functions with the highest contribution to the ordination model (highest scores), were progressively selected until the set of significant metabolic functions with the highest explanatory power, and lowest residual interference was reached (Borcard et al. 2011; Dinsdale et al. 2013). A generalized linear model (GLM) was used to test the statistical significance of the ordination scores of the first two components concerning the spatial distribution categories: clusterEgg, region, and sampled point. GLM model used a Gaussian distribution (F-test), verified through histogram and dispersion parameters (Borcard et al. 2011; Dinsdale et al. 2013).

## Results

### The microbial diversity of the mangroves

The metagenomic sequencing from eight mangroves in “Baía de Todos os Santos” (BTS) (Fig. 1 and Supplementary Table 1) yielded 1,717,124,604 raw reads. The samples CAM01MGA (from Cairu), JER02MGB (Jeribatuba), MAT04MGA (Mataripe), and NIT01MGA (Parque Niterói) generated very poor sequencing throughput and were removed from the analyses. A total of 1,594,536,856 high-quality reads were obtained from 92 samples, of which an average of 51.68% were assigned to a taxon (Supplementary Table 2).

**Fig. 2:**
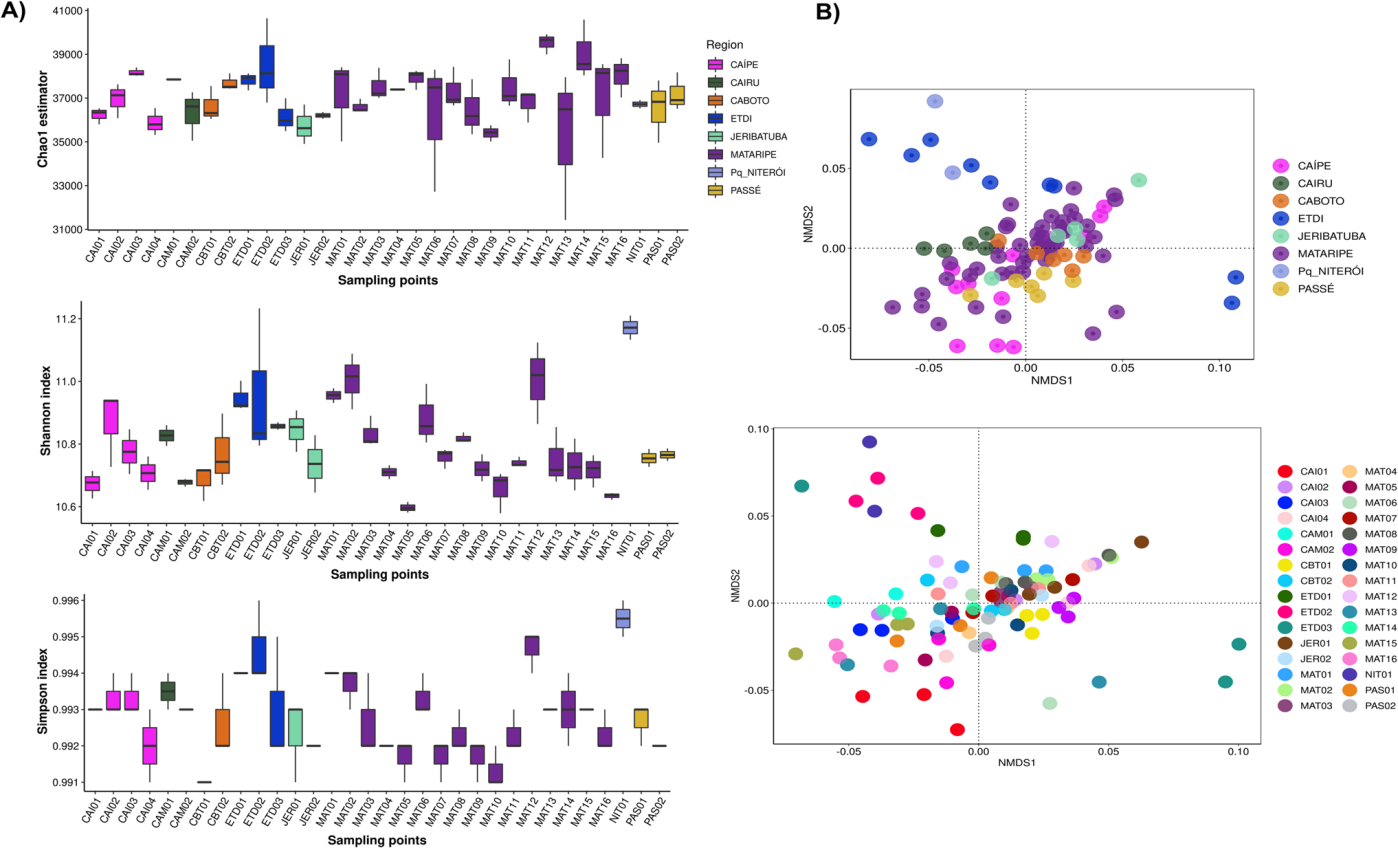
**A** Alpha diversity comparison of the bacterial compositions among the eight mangroves from the BTS area, measured according to the Chao1, Shannon, and Simpson indices. Boxes represent the interquartile ranges (IQRs) between the first and third quartiles (25th and 75th percentiles, respectively), and the line inside denotes the median. Whiskers indicate the lowest and the highest values within a range of 1.5-fold and the IQRs from the first and third quartiles, respectively. **B** Beta-diversity comparison between inter-studied regions and among the sampling points from each BTS mangrove region

The microbial richness and alpha diversity of the BTS mangrove sediment samples, grouped by region or sampling points, were measured by Chao1, Shannon, and Simpson indices (Fig. 2). Mangroves under refinery influence, such as NIT, ETDI, and MAT, showed the highest estimated richness compared to those described as a reference inside (JER) and outside the BTS (CAM) (Fig. 2A). Mangroves under refinery influence also showed the highest diversity index (Fig. 2A) (Supplementary Table 3). Caboto (CBT) and Passé (PAS), which suffer indirect effects of anthropogenic actions, showed higher species richness compared to JER and CAM (Fig. 2A). However, we observed a lower diversity in the anthropized mangroves (Fig. 2A). A heterogeneity of diversity was observed among sampling points, mainly for NIT, ETDI, and MAT (*p* < 0.05) (Supplementary Table 3). Shannon variations were significant for some comparisons between intra-regional replicates (Fig. 2A), such as for MAT and CAM (*p* < 0.05) (Supplementary Table 3). Interestingly, NIT showed high diversity (*p* < 0.05) (Supplementary Table 3), as well as high species dominance, even with only one sampling point (Fig. 2A). The beta diversity analysis revealed that NIT had a microbiota closer to that of ETDI and both have differences in species composition compared to the other samples (Fig. 2B).

### Microbial composition and differential abundance

The results show an abundance of taxa belonging to the bacterial phyla Thermodesulfobacteriota (Desulfobacteria class), Pseudomonadota (from Gamma- and Alphaproteobacteria class), Bacteroidetes (mainly from Bacteroidia and Flavobacteria class), and Planctomycetes (Phycisphaerae class), followed by the Actinobacteria and Chloroflexi phyla. The Cyanobacteria phylum was identified mainly in the ETDI region (Supplementary Fig. 1). Desulfobacteraceae, Rhodobacteraceae, Flavobacteriaceae, Halieaceae, and Chromatiaceae were the most representative families in the study area (Supplementary Fig. 2).

**Fig. 3:**
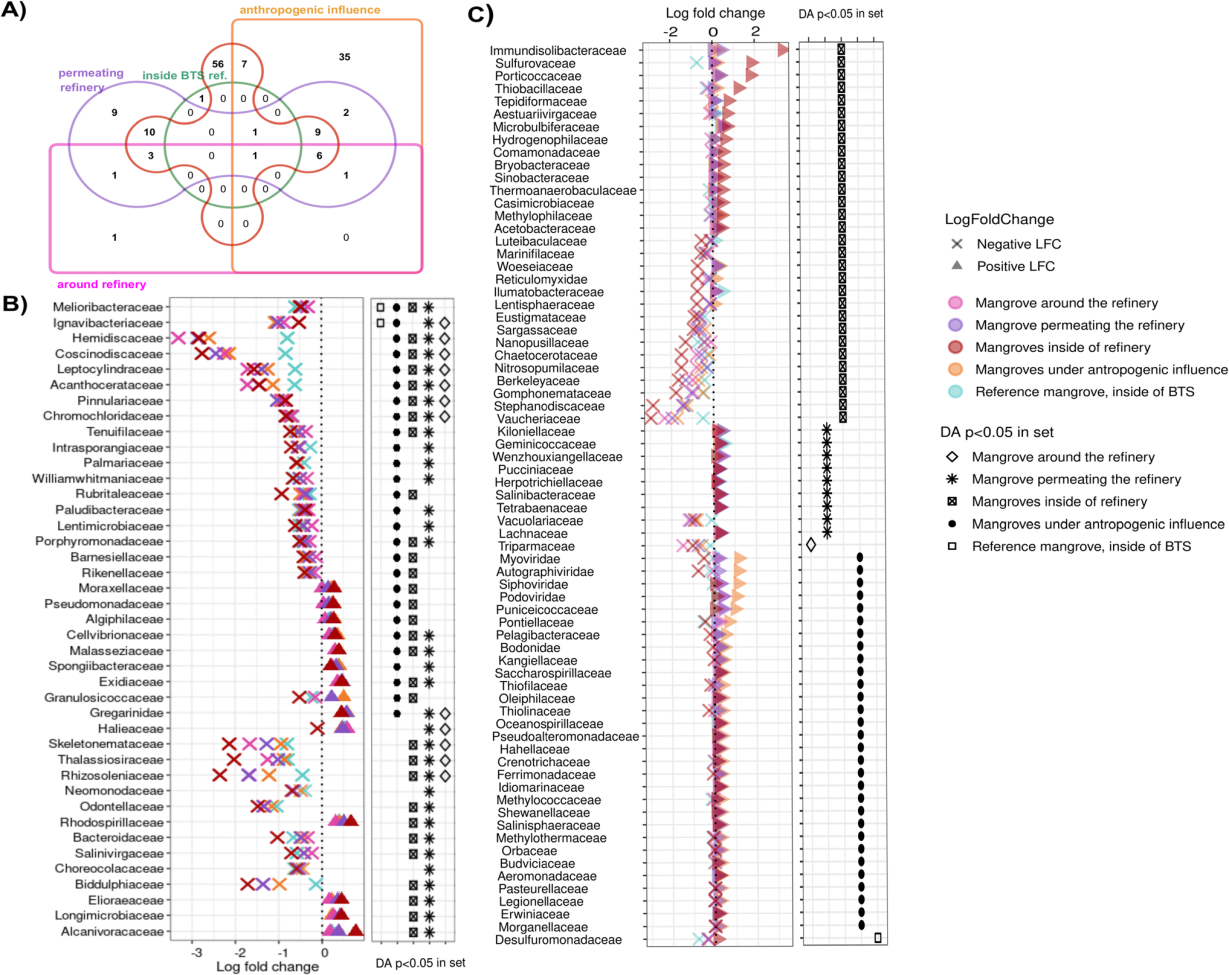
Differential abundance (DA) analysis of microbial composition identified in BTS mangroves for family taxonomic level. **A** Venn representation of common and exclusive families with significant DA in each studied area. **B** Representation of abundance variation of common families identified in the BTS mangroves, showing the highest log fold change (LFC) variations (positive and negative) for the area. **C** The highest LFC variations of families whose *p*-value showed DA significance only for one region. This analysis was conducted on disturbed mangroves compared to Cairu (CAM), the pristine mangrove, the reference outside the BTS. ETDI (ETD) and Parque Niterói (NIT) points were assigned as “inside refinery”; Caípe (CAI) as “around refinery”; Mataripe (MAT) points as “permeating refinery”; Caboto (CBT) and Passé (PAS) as those under “anthropogenic influence”; and Jeribatuba (JER) as “inside BTS ref.”

The differential abundance (DA) analyses showed that 41 families were shared among all mangroves (*p* < 0.05) (Fig. 3A, B, Supplementary Table 4). An increase in abundance variation (positive log fold change—LFC) of hydrocarbon-degrading Alcanivoracaceae and purple nonsulfur Rhodospirillaceae, both Pseudomonadota phylum, was observed in mangroves inside (ETDI and NIT, LFC 0.775 and 0.669, respectively) and permeating the refinery (MAT, LFC 0.379 and 0.504, respectively) (Fig. 3A, B). An apparent decrease of more than twofold in abundance (negative log fold change) of diatoms from distinct families was observed in different mangrove sets, such as Biddulphiaceae and Odontellaceae (in mangroves inside—ETDI and NIT; and permeating the refinery—MAT); and Rhizosoleniaceae, Skeletonemataceae, and Thalassiosiraceae (in mangroves around, permeating- and those inside- the refinery) (Fig. 3A, B). Compared to CAM, which is the preserved and referenced mangrove outside the BTS, all mangroves had a decreased abundance in Hemidiscaceae and Coscinodiscaceae diatoms families, less evidenced in JER, the reference inside the BTS (Fig. 3A, B).

Among the families present in one or more samples, but which differential abundance was significant for a specific sampling group, 56 were highlighted in ETDI and NIT (Fig. 3A and C, Supplementary Table 3). At the same time, 35 were significant for Caboto and Passé, both anthropized mangroves (Fig. 3A and C, Supplementary Table 4). For instance, ETDI and NIT had a significant abundance of bacterial families Immundisolibacteraceae (LFC 3.33) and Porticoccaceae (LFC 1.81) (both Gammaproteobacteria), as well as Sulfurovaceae (Epsilonproteobacteria) (LFC 1.84); and reduction of Vaucheriaceae and Stephanodiscaceae diatoms families (LFC − 2.96 and − 2.86, respectively). CBT and PAS highlighted the occurrence of families from the Caudovirales virus order and the Puniceicoccaceae bacteria (Fig. 3A and C, Supplementary Table 4).

Analysis at lower taxonomic levels, such as genus and species, showed that the majority of the genera found in the studied mangroves belong to the Bacteria domain, being the most abundant: *Gemmatimonas* (Gemmatimonadetes phylum); *Pseudomonas* (belonging to the Pseudomonadota phylum and Gammaproteobacteria class); *Streptomyces* (Actinomycetota phylum); *Halioglobus* (Pseudomonadota phylum and Gammaproteobacteria class), and Desulfosarcina (Thermodesulfobacteriota phylum and Desulfobacteria class) (Supplementary Fig. 3). Among the species, the *Gammaproteobacteria bacterium*, *Deltaproteobacteria bacterium*, *Chloroflexi bacterium*, *Chromatiales bacterium*, and *Acidobacteria bacterium* were the most abundant (Supplementary Fig. 4). A high intra-variability in microbial composition was observed at the genus and species levels among the sampling points of each region studied (Supplementary Figs. 3 and 4).

**Fig. 4:**
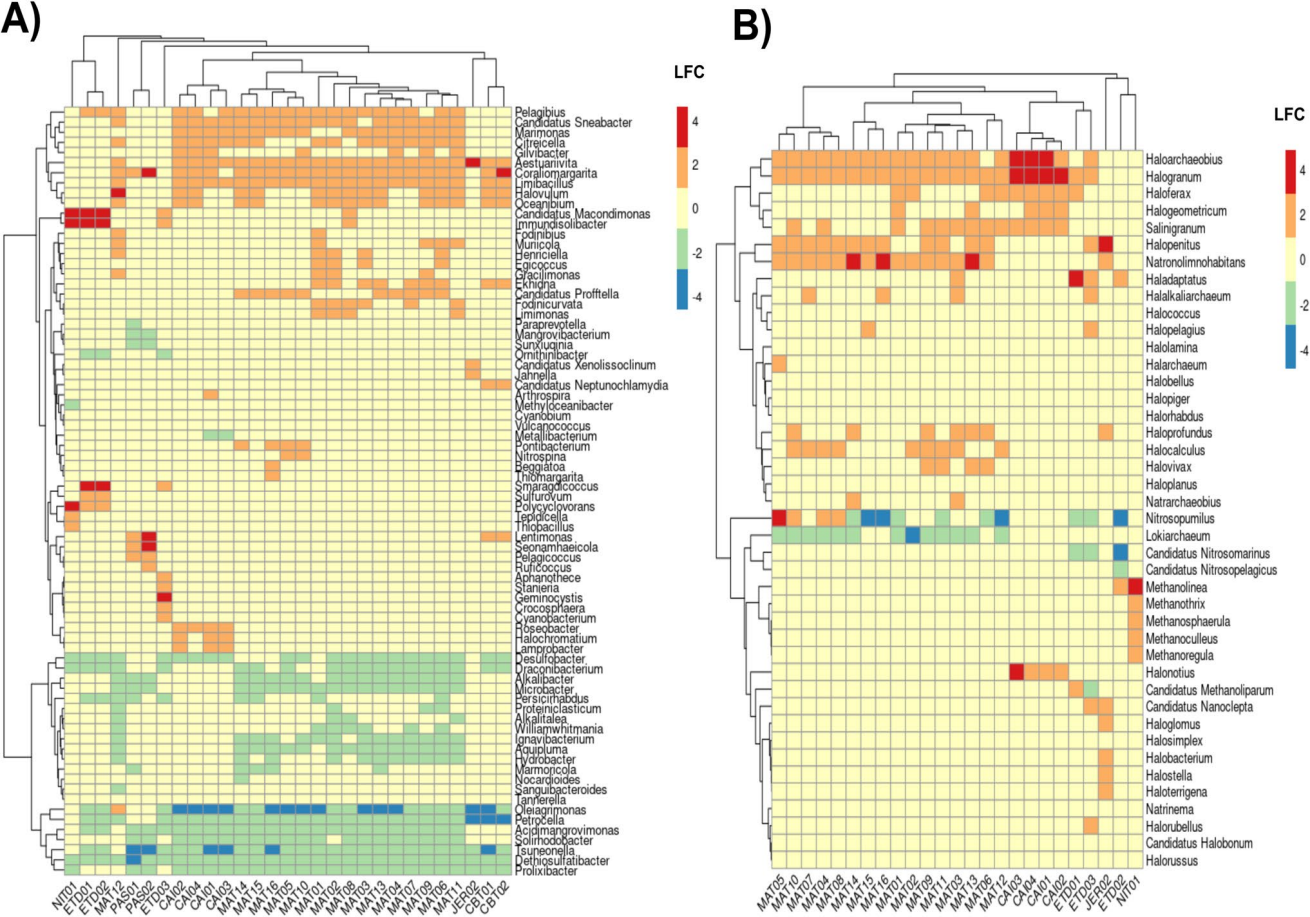
Differential abundance (DA) analysis of microbial composition identified in BTS mangroves at genus taxonomic level. **A** Genus with the highest log fold change values (positive and negative FLC) with *p*-value < 0.05 for the Bacteria domain. **B** Archaeal genus with the highest log fold change values (positive and negative FLC) with *p*-value < 0.05. No archaeal genus with significant DA was observed at Caboto (CBT) points. This analysis was conducted on mangroves inside the BTS, compared to Cairu (CAM), the pristine mangrove, and the reference outside the BTS. Points of each region were numbered, being those of ETDI designed as “ETD”; Caípe as “CAI”; Mataripe as “MAT”; Caboto as “CBT”; Jeribatuba as “JER,” and Passé as “PAS”

In the differential abundance analysis conducted for the Bacteria domain and comparing all mangroves to the CAM mangrove (reference outside of BTS) (Fig. 4A), the refinery-impacted mangroves (ETDI and NIT) showed *Immundisolibacter and Candidatus Macondimonas* genera with the highest abundance ratio variations (LFC > 4) between those differentially significant. Parque Niterói differed from ETDI by the abundance of PAH-degrading *Polyclyclovorans*, the genus related to sulfur oxidation *Thiobacillus*, and *Tepidicella.* In addition, ETDI has a predominance of actinomycete *Smaragdicoccus*, the Rhodospirillaceae member *Pelagibius*, and the sulfur-oxidizing bacteria *Sulfurovum.* Moreover, *Geminocystis*, a genus of Cyanobacteria, was highlighted in ETD03 (Fig. 4A).

In the anthropized areas of PAS and CBT, an increase in the abundance of *Coraliomargarita* and *Lentimonas* (both belonging to the Puniceicoccaceae family) was evidenced. JER, a reference inside the BTS, showed a high occurrence of *Aestuariivitta* (Rhodobacteraceae family) and a reduction in the abundance of *Petrocella* (Vallitaleaceae family) and *Oleiagrimonas* (Rhodanobacteraceae family) (LFC <  − 4) (Fig. 4A). Interestingly, *Oleiagrimonas* showed a positive log fold change in one point of MAT (MAT12). Mataripe, which permeates the refinery, showed a distinct profile of genera occurring in greater and lesser abundance than other areas. *Roseobacter*, *Halochromatium*, and *Lamprobacter* were abundant, mainly in the CAI area around the refinery. Otherwise, most sampling mangroves showed a reduction of sulfate-reducing *Desulfobacter* (LFC <  − 2), except in PAS and JER (Fig. 4A).

For Archaea, the CAI area showed an abundance of halophilic genera *Haloarchaeobius*, *Halogranum*, and *Halonotius* (Fig. 4B). A diminished abundance of ammonia-oxidizing *Nitrosopumilus* and *Candidatus Nitrosomarinus* was found in the majority of mangroves. Additionally, the methanogenic archaeon *Candidatus Methanoliparum* was reduced in the JER area and increased in ETDI (Fig. 4B).

### Metabolic structure and taxa-functional correlation in BTS mangroves

The metagenomic assembly generated around 122,830,790 contigs, with an average length of 400,803,833 bp. The N50 statistics showed that more than 50% of contigs were longer than 545,661 bp. Gene prediction resulted in 15,569,562 ORFs. The median of coding sequences varied from 6.58 to 18.97% per sample (Supplementary Table 2).

**Fig. 5:**
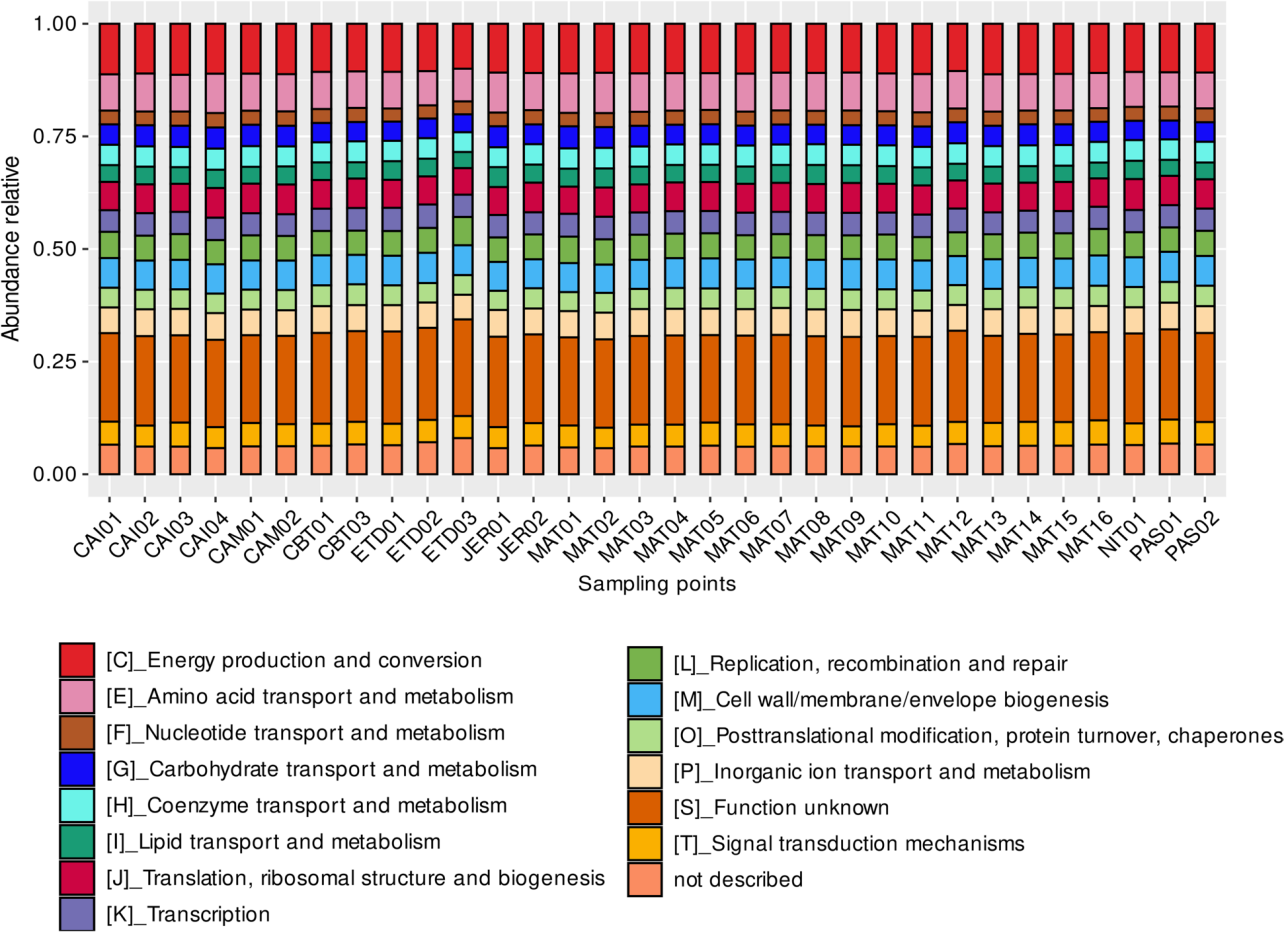
Relative abundance of the predominant metabolic categories in the points of BTS mangroves according to COG (Clusters of Orthologous Genes) from the EggNOG database

The annotation of ORFs according to the EggNOG database showed that the most abundant functional COG categories throughout the study area were energy production and conversion (C) (10.24%); amino acid transport and metabolism (E) (8.21%); cell wall/membrane/envelope biogenesis (M) (5.86%); translation, ribosomal structure, biogenesis (J) (5.58%); and transcription (K) (5.21%) (Fig. 5). Little functional variation was observed between sampling points. Of the 383 COG categories identified, 19% were common to all sampling points and triplicates and 36% occurred in at least 50% of the total 92 samples. Significant COG variations were found at the points: Caípe (CAI02, CAI04), Caboto (CBT02), ETDI (ETD02, ETD03), and Mataripe (MAT01, MAT02, MAT06, MAT07, MAT08, MAT11, MAT12, MAT13, and MAT15).

**Fig. 6:**
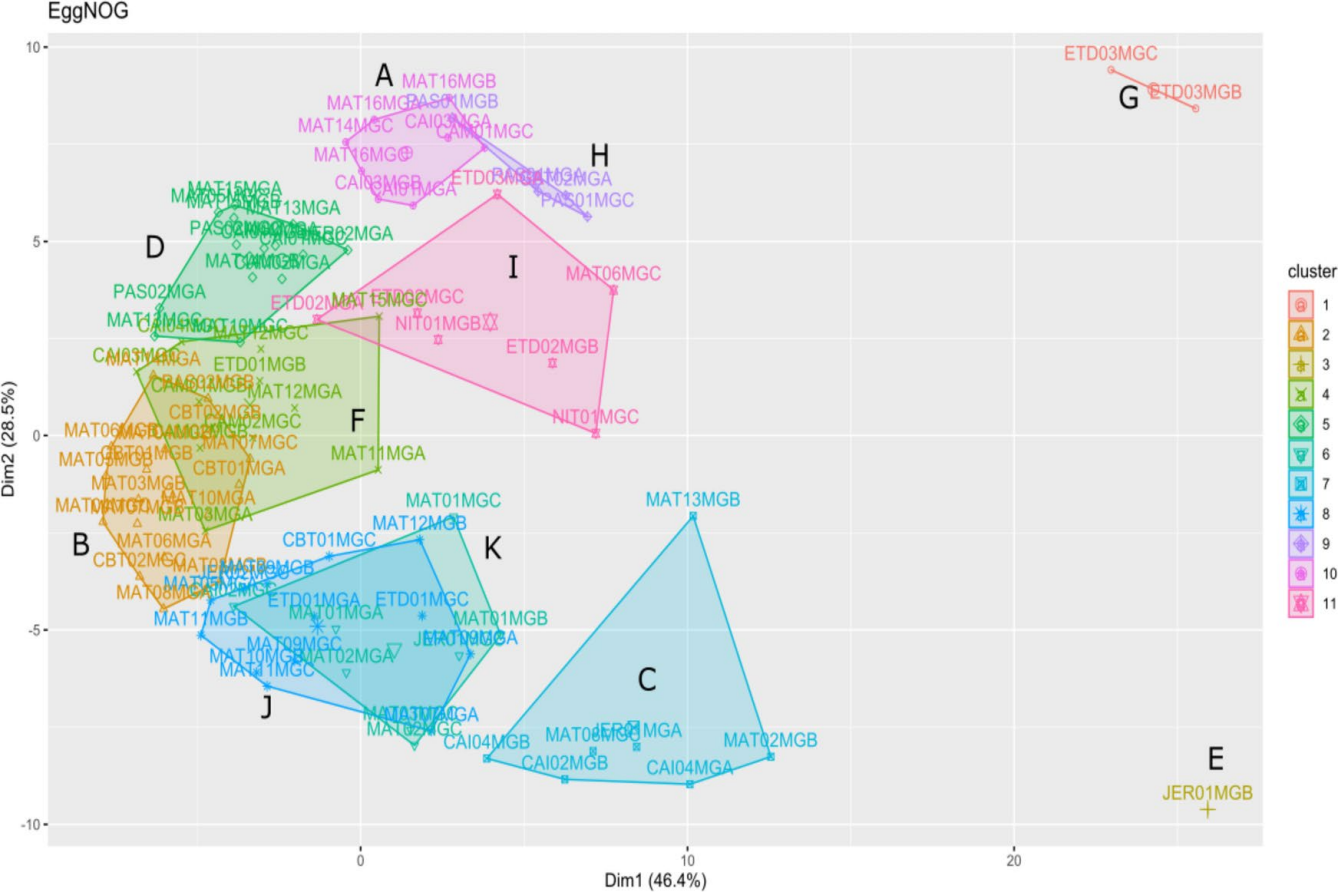
*K*-means clustering analysis of COG categories from the EggNOG database. According to the metabolic functions identified in the studied mangroves, the samples were grouped in 11 clusters (named A to K), each showing differences in COG categories

The *K*-means clustering partitioning method was adopted since a high heterogeneity among the triplicates variance was observed. The *k* = 11 showed the best clustering variance, with sample groupings more internally homogeneous and distinct from each other (Fig. 6). ETD points were the most metabolically diverse, generating two clusters, one composed of ETD03 triplicates (B and C) (cluster G) and another by ETD02 point plus ETD03 A (cluster I) (Fig. 6).

**Fig. 7:**
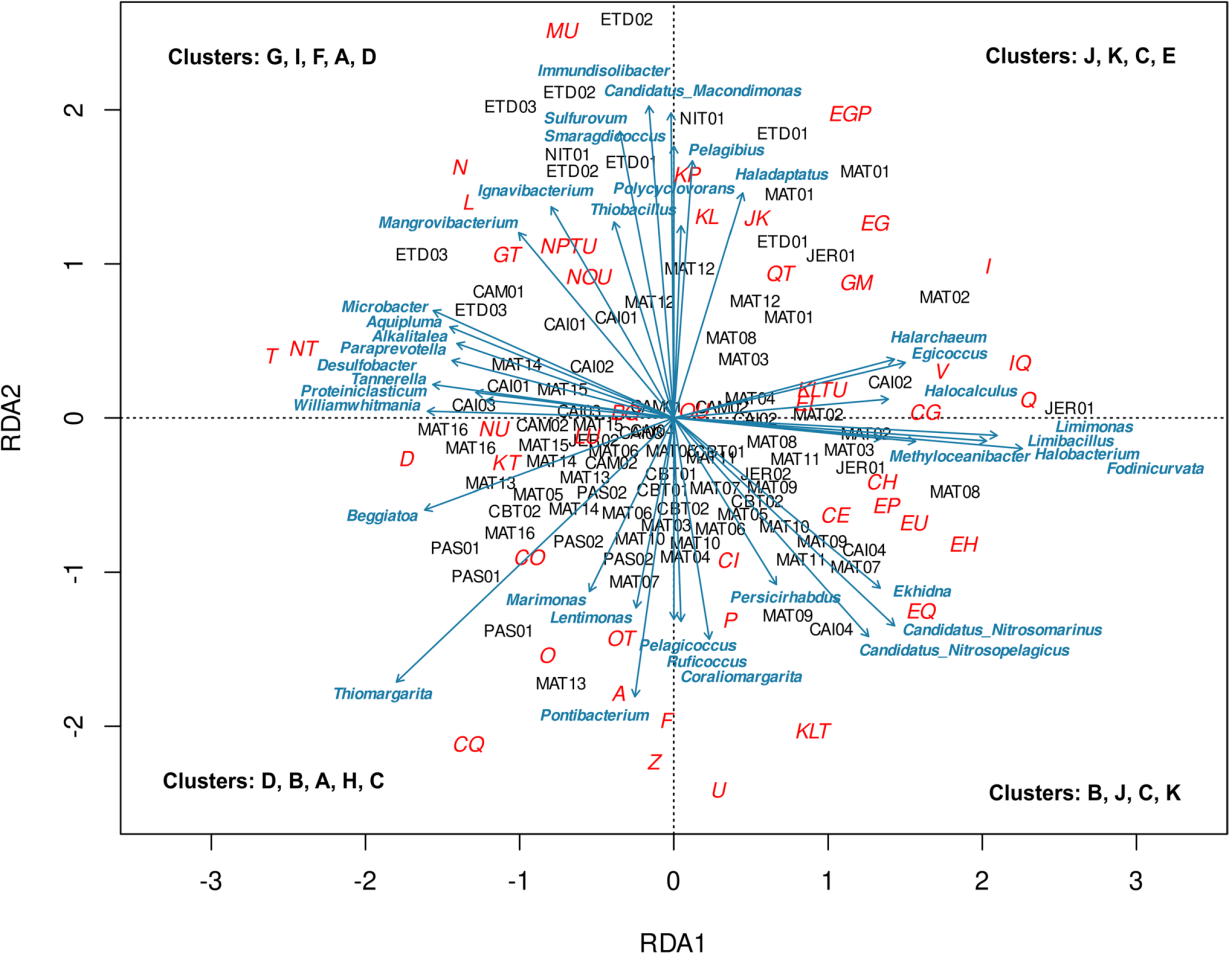
Redundancy analysis (RDA) to COG categories from the EggNOG database. Arrows show the strengths and contributions of variables to each *K*-means cluster. Sampling points are highlighted in black, COG categories in red, and taxonomic composition at the genus level in blue. The *K*-means groups are shown in bold

The distribution of functional categories significantly correlated to the sampling clusters and microbial taxa distribution (PERMANOVA *p* = 0.001) (Supplementary Table 5). The multivariate redundancy analysis (RDA) explained 35% of the total variance (RDA1 12%, RDA2 23%) and the highest metabolic contributions to each cluster are highlighted (Fig. 7).

The RDA upper left square contains mainly ETD and NIT points inside the refinery. The highest metabolic contributions to the ETD were cell motility (N) and replication, recombination, and repair (L) (for cluster G), while metagenomic sequences related to the membrane and intracellular trafficking secretion (MU) explained the greatest variations in both ETDI (ETD02 and ETD03) and NIT (cluster I). These functional groups were most strongly correlated to *Immundisolibacter*, *Candidatus Macondimonas*, and *Smaragdicoccus* hydrocarbon-degrading bacteria and also to sulfur-related bacteria *Sulfurovum.* Points of refinery-disturbed mangroves (ETDI—ETD03) around and permeating the refinery (Caípe—CAI01, CAI03, and Mataripe—MAT14 and MAT15) (clusters A, F, and D) were mainly associated with metabolic mechanisms involved in signal transduction mechanisms (T) and/or cell motility (NT, N), which are correlated to *Williamwhitmania* and *Desulfobacter* genera (Fig. 7).

At the RDA upper right square (Fig. 7), we observed two metabolic scenarios, one marked by central metabolism activity and another by biotransformation processes and bioconversion of organic and toxic components. In the first one, we highlighted the functions: amino acid transport and metabolism; carbohydrate transport and metabolism; inorganic ion transport and metabolism (EGP), mainly linked to the ETD01 point (cluster J). This correlated specifically with the point MAT01 of Mataripe (cluster K) by the contribution of metagenomic sequences related to amino acid and carbohydrate (EG) metabolism. These clusters showed a strong correlation with the occurrence of aerobic marine bacteria *Pelagibius* and the halophilic archaea genus *Haladaptatus.* Otherwise, in the second scenario, lipid transport and metabolism (I), secondary metabolites biosynthesis, transport and catabolism (Q), and defense mechanisms (V) were the main categories most correlated to the sampling points of Mataripe (MAT02, MAT03, MAT04, MAT08), Caipe (CAI02), and Jeribatuba (JER01) (clusters C and E). These variations were associated with halophilic microorganisms, such as the archaeal genera *Halarchaeum* and *Halocalculus*, and the bacterial genus *Egicoccus.*

The lower left square of RDA was composed of the majority of points from anthropized regions (Passé—PAS01 and PAS02; Caboto—CBT02) and the mangrove permeating the refinery (Mataripe—MAT13 and MAT16) (clusters A, B, and C). These regions were mainly associated with distinct metabolic mechanisms, such as energy production and conversion/secondary metabolites biosynthesis transport and catabolism (CQ); cytoskeleton (Z); and nucleotide transport and metabolism (F). These functional contributions were given mainly by the sulfur bacteria *Thiomargarita* and the moderately halophilic bacteria *Pontibacterium* (Fig. 7).

In some points from mangroves around and permeating the refinery (Caípe—CAI04 and Mataripe—MAT 07 and MAT09), at a lower right square of RDA, the occurrence of the bacterial genus *Fodinicurvata* was notary, as well as the archaeal *Candidatus Nitrosomarinus* and *Candidatus Nitrosopelagicus*, and the bacterial genus *Coraliomargarita* in lower contribution. These genera were associated with amino acid transport and metabolism; coenzyme transport and metabolism (EH); amino acid transport and metabolism, and intracellular trafficking, secretion, and vesicular transport (U).

## Discussion

The microbial community living in tropical and subtropical mangroves has an important role in the organic matter decomposition and the recycling of nutrients of mangroves, maintaining the high ecological diversity and productivity of these ecosystems (Holguin et al. 2001). Bacteria and fungi are the major constituents of total microbial biomass in tropical mangroves (Alongi 1988). The microbial metabolic activity decreases the nutrient-deficient of these habitats through different ecological functions, such as in the carbon cycle, nitrogen fixation, anoxygenic photosynthesis, phosphate-solubilization, and sulfate-reduction (Thatoi et al. 2013).

A systematic meta-analysis of published studies on microbial diversity of different mangrove conditions pointed out that the microbial composition of these soils is mostly sulfur-related (Lai et al. 2022). Despite the sulfate-reducing Desulfobacteraceae, the microbial composition of mangroves from Baía de Todos os Santos showed a predominance of members even previously described in other Brazilian mangroves, such as Flavobacteriaceae and Rhodobacteraceae. Flavobacteriaceae was reported in anthropized soil from Ceará and Bahia States, both located in the Northeastern region (Rocha et al. 2016a, b), while Rhodobacteraceae was abundant in disturbed mangroves from São Paulo State, Southeastern region (Andreote et al. 2012). Besides, the differential abundance analysis conducted for BTS sampling areas revealed distinct microbial profiles of those previously reported in mangroves from Brazil (Nogueira et al. 2015). The disturbed mangroves from BTS exhibited higher microbial diversity than preserved sampling areas, which was also observed by De Santana et al. (2021) and Lai et al. (2022). We found a wide diversity of sulfate- and sulfur-reduction bacteria in the mangroves from BTS. Taketani et al. (2010) showed that sulfate- and sulfur-reduction bacteria microorganisms are naturally selected in pristine mangrove sediments due to their usual deficit of oxygen and the abundance of organic matter, whose alteration significantly decreases the microbial diversity. This can explain the reduction of *Desulfobacter* in the BTS mangroves, particularly those located inside the refinery area, compared to the pristine mangrove (reference outside of BTS). Li et al. (2019) inferred that degradation of organic contamination triggered by sulfate-reducing conditions could contribute to the enrichment of sulfate-reducing bacteria as a possible effect of bioremediation in mangroves.

In anthropized mangroves, we highlighted biomass degradation species, with the occurrence of Puniceicoccaceae, a family belonging to the Verrucomicrobia phylum, ubiquitous in the ocean, and also of viruses from the Caudovirales order. *Coraliomargarita* spp. (a member of Puniceicococcaceae) was abundant in BTS. Doherty et al. (2017) have suggested that these microorganisms can be specialized in the degradation of plant biomass and exudates by containing genes for sulfatases, α-L-fucosidases, and β-agarases. Soil disturbances cause an increase in organic matter oxidation and a reduction of the soil structure by degrading the loss in soil aggregates (Punia and Bharti 2023), which can explain the elevated abundance of species involved in biomass degradation in anthropized mangroves here studied. Moreover, in cases of advanced disturbance, mangrove sediments can lose up to 80% of their potential to degrade/utilize carbon, decreasing the ability of the system to perform organic matter degradation and convert primary production into biomass (Carugati et al. 2018). Additionally, an uncharacterized diversity for Caudovirales virus was reported in mangrove soils, most of them phylogenetically distant to known reference sequences and formed three clades within Sipho- and Podoviridae families (Jin et al. 2019). Viruses can significantly influence local and global biogeochemical cycles, acting in carbon decomposition in mangroves by recycling complex polysaccharide (Jin et al. 2019; Cao et al. 2022). Lelchat et al. (2019) experimentally demonstrated that marine phages can display active polysaccharides in bacterial EPS, hypothesizing that viruses could also contribute to the degradation of marine organic matter and modify its bioavailability. The microbiota of mangroves around the refinery was characterized by the occurrence of halophilic and adapted to fluctuating salinity conditions genera belonging to the Chromatiaceae family (Caumette et al. 1988) and of wide distribution in various hypersaline environments (Kim et al. 2011; Mori et al. 2016). Mangroves from BTS that permeated the refinery were associated with extremely halophilic Archaea, which correlated to lipid metabolism. The lipid metabolism of halophilic Archaea has been related to the acquisition of precursors for membrane backbones, such as those derived from the catabolization of glycerol, which are abundantly produced by green algae in response to osmotic pressure (Falb et al. 2008). In the studied mangroves permeating the refinery, we also emphasized the presence of *Fodinicurvata*, a bacterial genus of Rhodospirillaceae family, described as hydroxybutyrate-producer, a biodegradable polyester (Wang et al. 2009).

BTS mangroves inside and permeating the refinery, on the other hand, showed a wide and elevated occurrence of the hydrocarbon degradation microorganisms, especially of Gammaproteobacteria (such as a member of the Alcanivoraceae family and members of the genera: *Immundisolibacter*, *Candidatus Macondimonas*, *Polycyclovorans*, *Oleiagrimonas*), the Burkholderiales *Tepidicella*, and the Actinomycete *Smaragdicoccus.*

Members of the Alcanivoracaceae family, when in unpolluted areas, can consume the natural hydrocarbons, such as isoprene, produced by marine phytoplankton (Coulon et al. 2012). *Immundisolibacter cernigliae*, the single described species of the genus, was isolated from aerobic bioreactor-treated soil from the USA and is associated with PAH degradation (Corteselli et al. 2017). Representatives of the *Candidatus Macondimonas* have been described as highly abundant in contaminated coastal marine sediments and play a key ecological role in response to global oil spills (Karthikeyan et al. 2019). Additionally, the genus *Polycyclovorans* contains species isolated from marine diatoms and enrichment in the presence of aromatic hydrocarbons (Gutierrez et al. 2013). *Oleiagrimonas*, a genus reported in oil-polluted saline soil, contains species whose extracellular polymeric substances may contribute to bioremediation (Huang et al. 2015).

The predominance of hydrocarbon degradation microorganisms found in the BTS mangroves under the influence of the refinery is mainly associated with defense and secretion mechanisms. Cell motility is an important adaptation of heterotrophic bacteria and archaea to exploit resources, enhance bacteria-organic-matter coupling, and escape from unfavorable conditions (Grossart et al. 2001). Curiously, *Williamwhitmania*, which was associated with our data on ETDI region and the functional category cell motility, has an unusual gliding motility by external organelles (Pikuta et al. 2017). Secretion of extracellular polymeric substances (EPS) is another strategy of microbial defense. Their positive influence on the bioremediation process against the PAH contamination was demonstrated by Ali et al. (2022). The EPS secretion coincides with an increase in reactive oxygen production, which accumulation could cause damage to biomolecules and be lethal to marine species (Morris et al. 2022).

Chen et al. (2018) argue the deleterious effects of petroleum exploration, with a significant increase of PAH and total organic carbon after the oil production and construction of the Mataripe refinery (1939–1950). However, features of mangroves such as high productivity, abundant detritus, and rich organic carbon make it a preferential site for the uptake and accumulation of pollutants such as PAHs (Bernard et al. 1996). The oxidizing and mineralizing of hydrocarbons by microorganisms strongly selected in impacted environments could then act to prevent the PAH-accumulation in the sea (Duran and Cravo-Laureau 2016) and their genotoxic effects in marine sediment species (Nikolaou et al. 2009; Munoz and Albores 2011), contributing to the restoration of the mangroves.

In August 2019, the Brazilian Northeast was the target of a large-scale environmental disaster due to oil spill contamination, including the state of Bahia and the surroundings of Bahia de Todos os Santos (IBAMA 2020; Silva et al. 2021). Our data allowed us to assess the microbial community and biological processes of mangroves from Baía de Todos os Santos, Brazil, as an investigative overview of the protection status of these estuaries, especially of those disturbed areas. The anthropized estuaries showed species related to biomass degradation associated with central metabolism pathways. Differentially from pristine and anthropized areas, BTS mangroves under the influence of the refinery presented a species enrichment indicative of impacted sediment, despite the limitation of soil properties conditions. These specialized microbiota, notably distinct and not previously abundant in Brazilian estuarine soils, are adapted to the PAH increase and are highly associated with cell signaling processes. These data suggest that these species are in an active process of responding and adapting to the environment, contributing to the recovery of disturbed mangroves. Finally, our data point that metagenomics can be used as an additional and predictive environmental assessment tool and reinforce the need for constant monitoring practices to preserve the Brazilian mangroves.

## Supplementary Information

Below is the link to the electronic supplementary material.

Supplementary file1 (PDF 1546 KB)

Supplementary file2 (XLSX 68 KB)

Supplementary file3 (XLSX 60 KB)

Supplementary file4 (XLSX 123 KB)

Supplementary file5 (XLSX 215 KB)

Supplementary file6 (XLSX 103 KB)

## Data Availability

The SRA files of metagenomic raw paired-end reads for this study were deposited at the National Center for Biotechnology Information (NCBI) (https://www.ncbi.nlm.nih.gov/), under accession number PRJNA954358.
