# Supplementary material for: Finding microbial composition and biological processes as predictive signature to access the ongoing status of mangrove preservation
Source: Int Microbiol. 2024 Feb 22;27(5):1485–500. doi: 10.1007/s10123-024-00492-z (PMC11452435; doi:10.1007/s10123-024-00492-z)
Supplement: Supplementary file 1 — Supplementary file1 (PDF 1546 KB) [file 10123_2024_492_MOESM1_ESM.pdf]

Manuscript "**Finding microbial composition and biological processes as predictive signature to access the ongoing status of mangroves preservation**". International Microbiology. Fabíola Marques de Carvalho et al. Corresponding author: Ana Tereza R. de Vasconcelos - Laboratório de Bioinformática, Laboratório Nacional de Computação Científica, Petrópolis, Brazil - email: email: atrv@lncc.br

SUPPLEMENTARY FIGURES

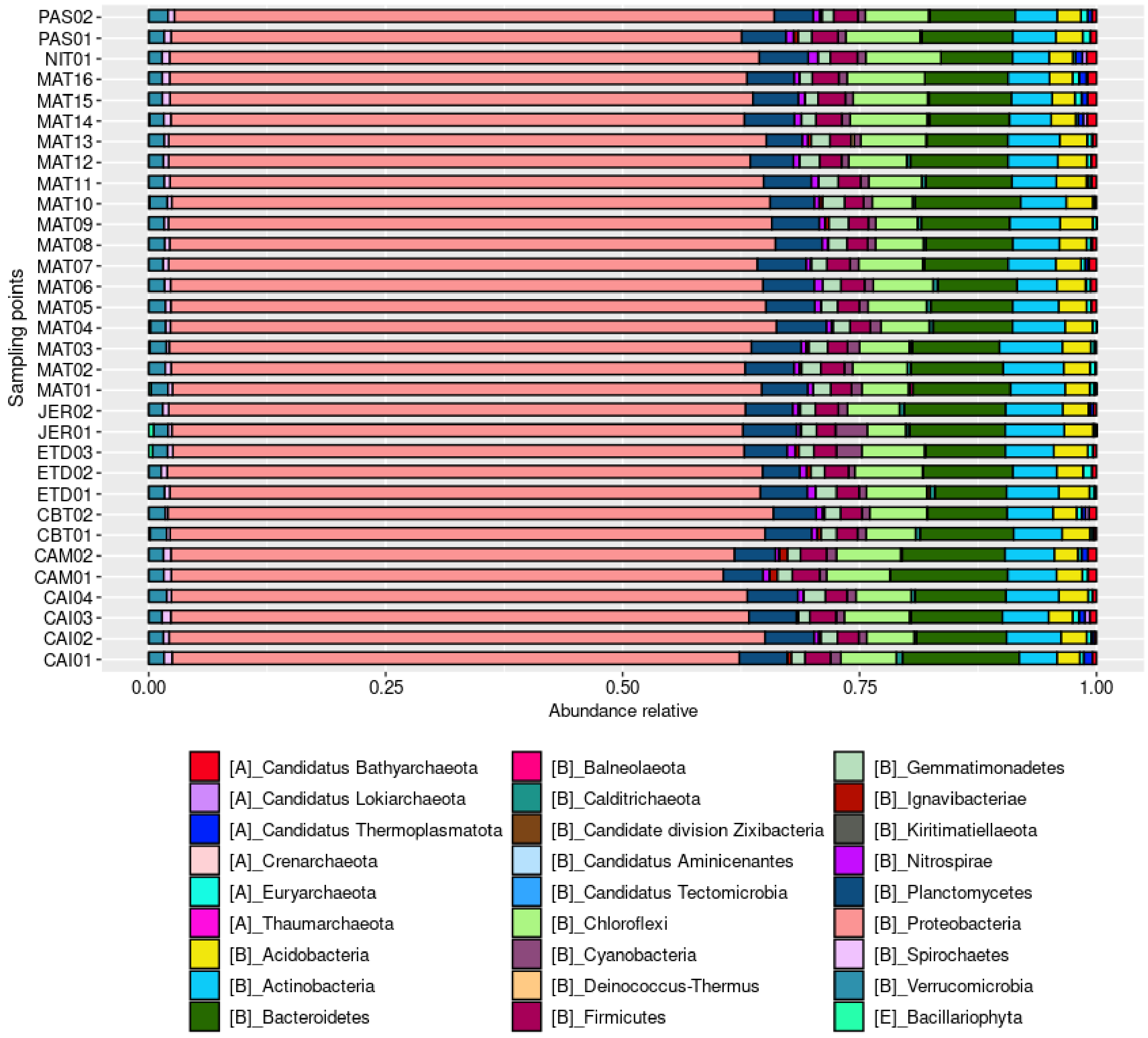

Supplementary Fig. 1 Relative frequency of 15 predominant phyla identified in each sampling mangrove point. [A] denotes that the phylum belongs to the Archaea domain; [B] from the Bacteria domain, and [E] those from Eukarya domain. Viruses were identified in samples, however, they are not among the 15 most abundant phyla.

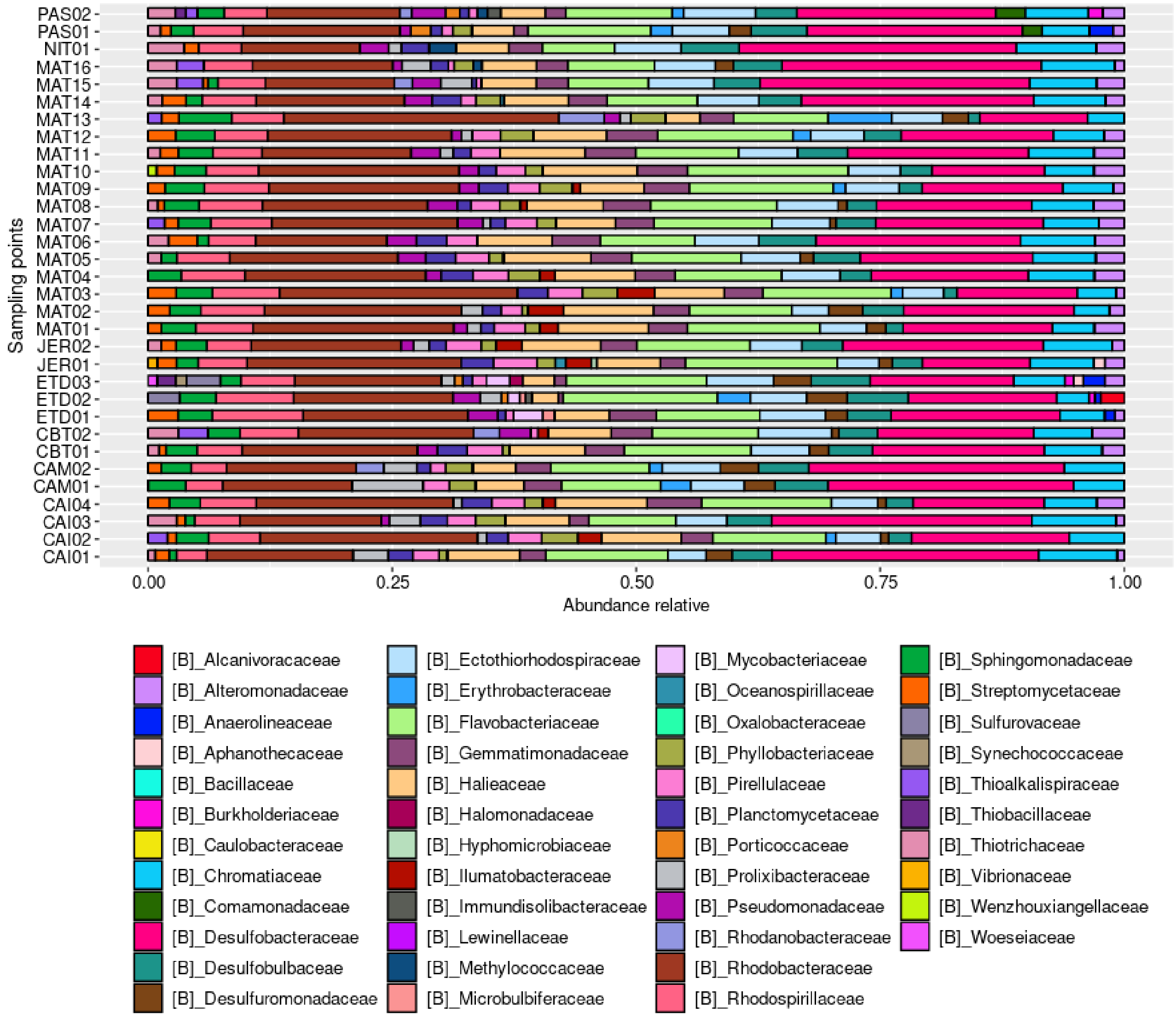

Supplementary Fig. 2 Relative frequency of predominant families identified in each sampling mangrove point. [B] denotes that the family belongs to the Bacteria domain. Families from Archaea, Eukaryota, and also Viruses are not among those 15 most abundant.

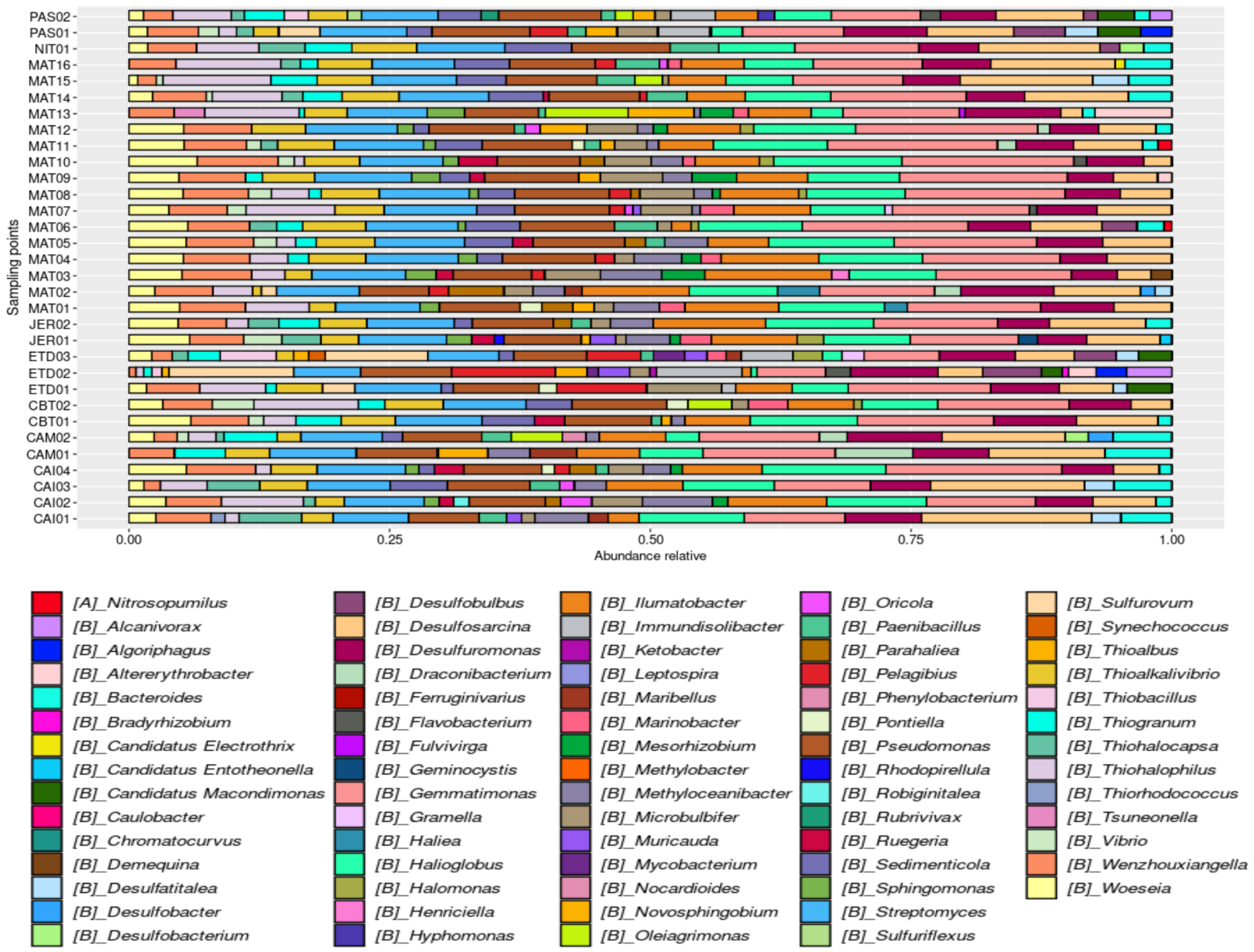

Supplementary Fig. 3 Relative frequency of predominant genera identified in each sampling mangrove point. [A] denotes that the genus belongs to the Archaea domain and [B] from the Bacteria domain. Eukarya and Viruses are not among the 15 most abundant genera.

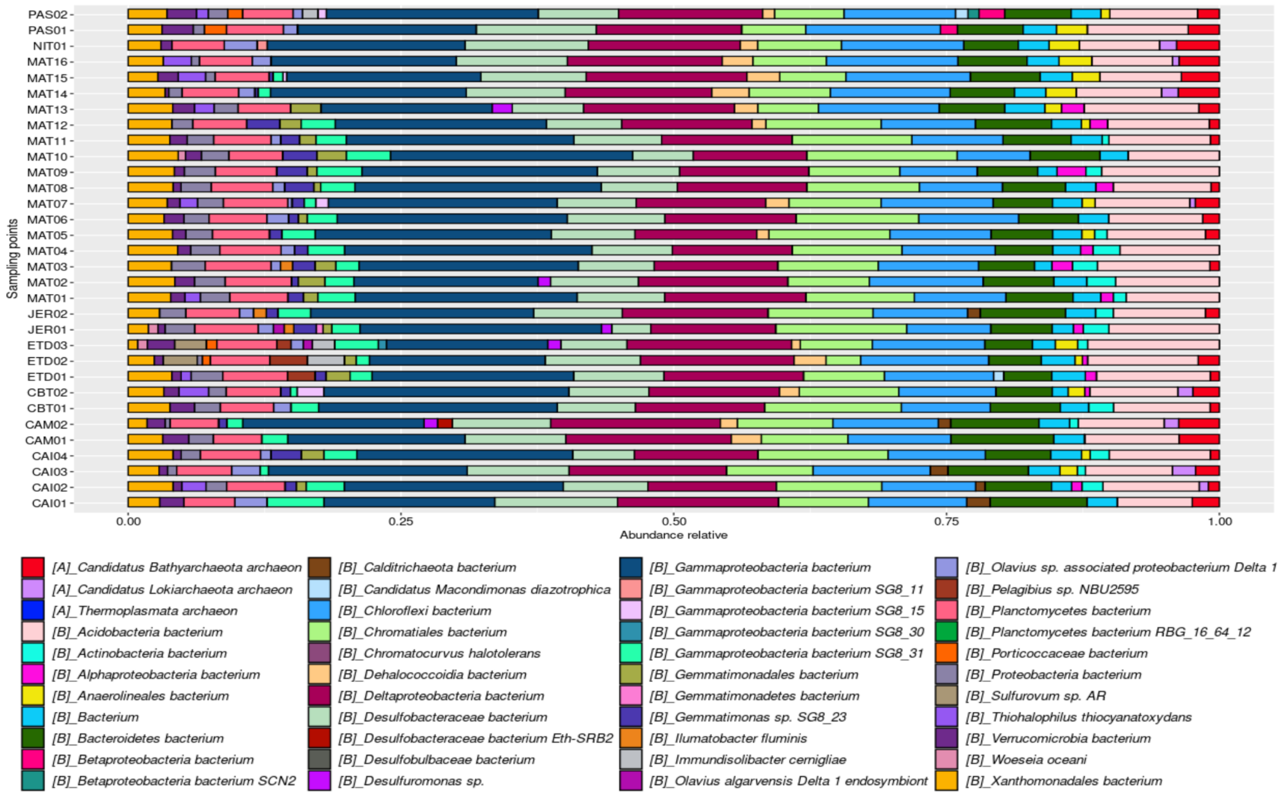

Supplementary Fig. 4 Relative frequency of 15 predominant species identified in each sampling mangrove point. [A] denotes that the species belongs to the Archaea domain and [B] from the Bacteria domain. Eukarya and Viruses are not among those 15 most abundant species.
